# Are polypharmacy side effects predicted by public data still valid in real-world data?

**DOI:** 10.1016/j.heliyon.2024.e24620

**Published:** 2024-01-17

**Authors:** Gaeun Kee, Hee Jun Kang, Imjin Ahn, Hansle Gwon, Yunha Kim, Hyeram Seo, Heejung Choi, Ha Na Cho, Minkyoung Kim, JiYe Han, Seohyun Park, Kyuwoong Kim, Tae Joon Jun, Young-Hak Kim

**Affiliations:** a Department of Information Medicine, Asan Medical Center, 88, Olympicro 43gil, Songpagu, 05505, Seoul, Republic of Korea; b Department of Medical Science, Asan Medical Institute of Convergence Science and Technology, Asan Medical Center, University of Ulsan College of Medicine, 88, Olympicro 43gil, Songpagu, 05505, Seoul, Republic of Korea; c Division of Cardiology, Asan Medical Center, 88, Olympicro 43gil, Songpagu, 05505, Seoul, Republic of Korea; d Big Data Research Center, Asan Institute for Life Sciences, Asan Medical Center, University of Ulsan College of Medicine, 88, Olympicro 43gil, Songpagu, 05505, Seoul, Republic of Korea; e Division of Cardiology, Department of Information Medicine, Asan Medical Center, University of Ulsan College of Medicine, 88, Olympicro 43gil, Songpagu, 05505, Seoul, Republic of Korea; f National Cancer Control Institute, National Cancer Center, 323 Ilsan-ro, Ilsandong-gu, 10408, Goyang, Republic of Korea

**Keywords:** Real-world data, Drug-drug interaction, Polypharmacy, Electronic health records, Retrospective study

## Abstract

**Background and Objective:**

Although interest in predicting drug-drug interactions is growing, many predictions are not verified by real-world data. This study aimed to confirm whether predicted polypharmacy side effects using public data also occur in data from actual patients.

**Methods:**

We utilized a deep learning-based polypharmacy side effects prediction model to identify cefpodoxime-chlorpheniramine-lung edema combination with a high prediction score and a significant patient population. The retrospective study analyzed patients over 18 years old who were admitted to the Asan medical center between January 2000 and December 2020 and took cefpodoxime or chlorpheniramine orally. The three groups, cefpodoxime-treated, chlorpheniramine-treated, and cefpodoxime & chlorpheniramine-treated were compared using inverse probability of treatment weighting (IPTW) to balance them. Differences between the three groups were analyzed using the Kaplan-Meier method and Cox proportional hazards model.

**Results:**

The study population comprised 54,043 patients with a history of taking cefpodoxime, 203,897 patients with a history of taking chlorpheniramine, and 1,628 patients with a history of taking cefpodoxime and chlorpheniramine simultaneously. After adjustment, the 1-year cumulative incidence of lung edema in the patient group that took cefpodoxime and chlorpheniramine simultaneously was significantly higher than in the patient groups that took cefpodoxime or chlorpheniramine only (p=0.001). Patients taking cefpodoxime and chlorpheniramine together had an increased risk of lung edema compared to those taking cefpodoxime alone [hazard ratio (HR) 2.10, 95% CI 1.26–3.52, p<0.005] and those taking chlorpheniramine alone, which also increased the risk of lung edema (HR 1.64, 95% CI 0.99-2.69, p=0.05).

**Conclusions:**

Validation of polypharmacy side effect predictions with real-world data can aid patient and clinician decision-making before conducting randomized controlled trials. Simultaneous use of cefpodoxime and chlorpheniramine was associated with a higher long-term risk of lung edema compared to the use of cefpodoxime or chlorpheniramine alone.

## Highlights

• Validating polypharmacy side effect predictions with real-world data informs patient and clinician decision-making.
• Concurrent use of cefpodoxime and chlorpheniramine raises lung edema risk compared to each drug alone.
• Utilizing real-world data is essential for improving adverse event prediction models' capability.

## Graphical abstract

**Figure:**
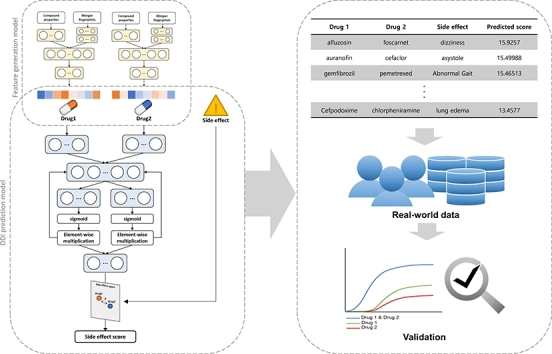

## 1 Introduction

Real-world data (RWD) refers to data related to patient health status or healthcare delivery that is routinely collected from various sources, including electronic health records (EHRs) [1]. Due to the limitations of existing clinical trials, such as high costs and small patient populations, research using RWD accumulated in hospitals is actively being conducted to bridge the gap between clinical research and actual data [2], [3], [4], [5], [6]. Although RWD also has limitations like potential issues with information quality, confounding variables, and bias, real-world evidence (RWE) may be more reliable and usable than randomized controlled trials (RCT) in detecting side effects. RWE is considered acceptable when it is comparable to the treated group, the association between drug exposure and the outcome is observable or measurable, major confounding variables are observable or measurable, and ethical and clinical RCTs are not feasible [7], [8], [9]. The Food and Drug Administration (FDA) acknowledges the importance of RWD in monitoring post-market safety and adverse events, as well as in making regulatory decisions [10].

With the increasing prevalence of polypharmacy, the concern for drug-drug interactions and their potential side effects has also grown [11], [12]. While combining two or more drugs may offer synergistic benefits like increased efficacy [13], it can also lead to unintended consequences if drug-drug interactions are not well understood [14]. Given the vast number of possible drug combinations, conducting clinical trials for each is impractical. Therefore, researchers have attempted to predict interactions between multiple drugs using machine learning.

Several prediction models have been developed to identify unknown side effects of multiple drug usage, including predicting polypharmacy drug response using graph convolutional network and graph feature attention network. Decagon is a convolutional graph neural network that predicts drug pairs side effects by constructing a two-layer multimodal graph of protein-protein interactions, drug-protein interactions, and drug-drug interactions [15]. The graph feature attention network (GFAN) model provides interpretable predictions of polypharmacy side effects by emphasizing target genes differently [16]. Additionally, the DeSIDE-DDI model uses molecular drug response signals to capture drug-drug interaction (DDI) mechanisms [17]. Despite their promising performance, few studies have validated these polypharmacy side effect prediction models in a population-based setting.

In this study, we utilized the DeSIDE-DDI model, a deep learning-based algorithm for predicting adverse effects of polypharmacy. We extracted unknown drug-drug-side effect combinations in order of the highest prediction scores. After confirming combinations with an appropriate number of patients and clinical significance for retrospective investigation, we selected the cefpodoxime-chlorpheniramine-lung edema candidate pair. Despite the prediction that absence of side effects when both drugs were co-administered, instances of side effects were observed in actual patient data. This emphasizes the significance of real-world patient data in validating the adverse event prediction algorithm for polypharmacy using artificial intelligence.

## 2 Methods

### 2.1 Selection of polypharmacy side effect prediction model

Among various drug-drug interaction prediction models, the DeSIDE-DDI method was chosen. This model has a clear structure and demonstrates higher accuracy compared to other models. Its overall performance was evaluated using various datasets such as BioSNAP and DrugBank [17]. Therefore, it was expected that the results predicted by this model would be highly relevant when verified using actual patient data.

### 2.2 Prediction of polypharmacy side effect

**Figure 1:**
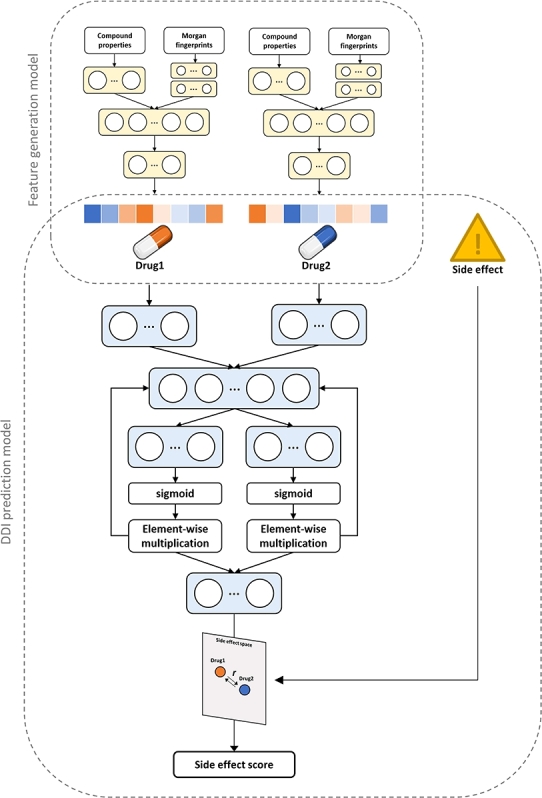
Overview of DeSIDE-DDI.

The DeSIDE-DDI is a model composed of two types of models: the feature generation model and the DDI prediction model. The overview of the drug-drug interaction prediction model is illustrated in Fig. 1.

The feature generation model outputs drug-induced gene expression when compound structures and property information are inputted. The datasets are level-5 data of LINCS L1000. The feature generation model is composed of simple neural networks, and drug fingerprints and properties are inputted into separate dense layers, which are then combined to provide predicted gene expressions.

The DDI prediction model utilized 4,576,287 drug-drug-side effect interactions between 63,472 drug combinations and 963 side effects using BioSNAP data refined from TWOSIDES drug information. The drug-drug-side effect pairs format was inputted into the DDI prediction model. The outputs of the feature generation model are represented by the drug features, which are applied as gated linear units for information control. The drug features are embedded into the side-effect space, and a distance is calculated drug-drug pairs.

The model was run under the same conditions and dataset as the unseen-unseen drug interaction prediction study conducted in the DeSIDE-DDI study, and the output drug-drug-side effect pairs were used in the experiments.

### 2.3 Study design and setting

We used the DeSIDE-DDI model to generate a list of drug-drug-side effect pairs, sorted by the highest prediction score. Subsequently, we extracted a list of drug-drug-side-effect pairs associated with unknown side effects. To determine the prevalence of these drug combinations among patients, we examined the usage records of both drugs at Asan Medical Center. Through discussions with clinicians, among the pairs with high prediction scores, the only pair exhibiting both clinically meaningful results and a statistically significant number of patients over 20 years of patient data was the cefpodoxime-chlorpheniramine-lung edema combination. Consequently, we conducted RWD-based validation for the cefpodoxime-chlorpheniramine-lung edema combination.

Initially, a total of 268,182 patients who had taken cefpodoxime or chlorpheniramine from January 2000 to December 2020 were included in a retrospective cohort study at the Asan Medical Center in Seoul, Korea. After applying the inclusion criteria of being ≥ 18 years old and taking medications orally, 259,568 patients were included in the study population. Patients who had pulmonary edema or pneumonia prior to taking cefpodoxime and chlorpheniramine were excluded from the study. The patients were followed for 20 years or until the first occurrence of any study outcome from the date of enrollment. Patients who were lost to follow-up were included in the analysis until the last follow-up period within 20 years or until the first occurrence of any study outcome from the date of enrollment. This study received independent approval from the Institutional Review Boards of Asan Medical Center (IRB no. 2021-0303).

### 2.4 Selection of participants

**Figure 2:**
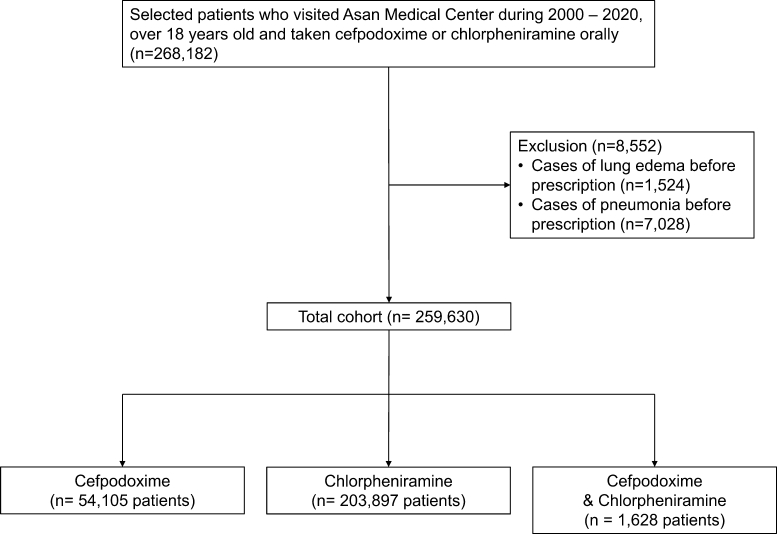
Flow chart.

**Figure 3:**
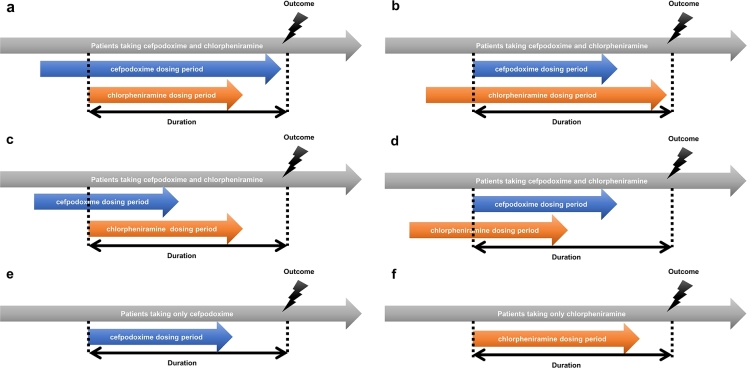
Duration of medications, the dosing period of the cefpodoxime & chlorpheniramine-treated group is shown as a, b, c, d, the dosing period of the cefpodoxime-treated group is shown as e and the dosing period of the chlorpheniramine-treated group is shown as f. The number of patients in each case is below. a=362; b=352; c=563; d=818; e=55,524; f=210,563.

Among patients who visited the Asan Medical Center, the first dose of cefpodoxime or chlorpheniramine was administered from January 1, 2000, to December 31, 2020. The patients were aged 18 years or older and had taken both medications orally, as recorded in ABLE [18], the patient database of Asan Medical Center. Of the two medications, 55,524 patients had taken cefpodoxime, 210,563 patients had taken chlorpheniramine, and 2,095 patients had taken both. Among them, 157 patients in the cefpodoxime patient group, 1,318 patients in the chlorpheniramine patient group, and 49 patients in the drug combination group had lung edema symptoms before starting treatment. Additionally, 1,262 patients in the cefpodoxime patient group, 5,348 patients in the chlorpheniramine patient group, and 418 patients in the drug combination groups had pneumonia symptoms before starting treatment. Excluding these cases, a total of 54,043 patients in the cefpodoxime patient group, 203,897 patients in the chlorpheniramine patient group, and 1,628 patients in the drug combination patient group were included in the experiment (Fig. 2). The conditions of patients who took cefpodoxime and chlorpheniramine simultaneously are presented in Fig. 3. The point at which the duration of taking cefpodoxime and chlorpheniramine overlapped was set as the starting date for simultaneous administration. For the cefpodoxime patient group, the first dose of cefpodoxime was used as the starting date, and for the chlorpheniramine patient group, the first dose of chlorpheniramine was used as the starting date.

### 2.5 Outcome measures

In this study, the DeSIDE-DDI model predicted lung edema as the polypharmacy side effect of cefpodoxime and chlorpheniramine. The definition of lung edema included cardiogenic pulmonary edema, noncardiogenic pulmonary edema, acute pulmonary edema, and chronic pulmonary edema. The ‘Concept id’ was ‘C0034063’, ‘SNOMED_CT’ was ‘19242006’, and ‘ICD-10 code’ was ‘J81’, ‘I50.1’. The outcome was defined as the first date of diagnosis for lung edema (ICD-10 codes: ‘J81’, ‘I50.1’) at Asan Medical Center from January 1, 2000, to December 31, 2020. The follow-up period was up to 20 years for patients. Patients who developed lung edema symptoms after starting concurrent administration of cefpodoxime or chlorpheniramine were considered to have an outcome. In the cefpodoxime and chlorpheniramine patient group, patients who developed lung edema symptoms after treatment were also considered as having an outcome.

### 2.6 Statistical analysis

To balance the three groups, an inverse probability of treatment weighting (IPTW) was used, utilizing propensity score-weighting [19], [20]. Categorical variables were compared Pearson's chi-square test, and a continuous variable was compared using one-way ANOVA. Descriptive characteristics of the study population are provided as number (%) for categorical variables and mean (SD) for a continuous variable. Kaplan-Meier curves for clinical outcomes were assessed by log-rank test, and Cox proportional hazards regression was used to estimate hazard ratios (HRs) for clinical outcomes. Potentially relevant variables, such as age, sex, hypertension, type 2 diabetes, cardiovascular disease, and pneumonia were included in the analysis. Data extraction and statistical analyses were performed using the “survival,” “jskm,” and “epiR” packages in R software version 4.2.2. (R Core Team (2022). R: A language and environment for statistical computing. R Foundation for Statistical Computing, Vienna, Austria. URL https://www.R-project.org/.)

## 3 Results

### 3.1 A selected drug-drug-side effect pair

Table 1 displays the top 15 scores of drug-drug-side effect pairs, which were not previously identified as polypharmacy side effects. From this list, we selected the cefpodoxime-chlorpheniramine combination, considering both clinical significance and a statistically significant number of patients in both the control and experimental groups among pairs with high prediction scores through discussions with clinicians.

**Table 1:** Predicted drug-drug-side effect pairs.

Rank	Drug1 Name	Drug2 Name	Side Effect ID	Side Effect Name	Predicted Score	Predicted Label
1	alfuzosin	foscarnet	C0012833	dizziness	15.9257	0
2	auranofin	cefaclor	C0018790	asystole	15.49988	0
3	gemfibrozil	pemetrexed	C0575081	Abnormal Gait	15.46513	0
4	dicyclomine	methyldopa	C0015230	eruption	14.56787	0
5	cerivastatin	azathioprine	C0497247	arterial pressure NOS increased	14.39701	0
6	rifaximin	BCNU (carmustine)	C0085624	burning sensation	13.963	0
7	dicyclomine	busulfan	C0151766	Abnormal LFTs	13.75256	0
8	cefprozil	oxandrolone	C0231218	Feeling unwell	13.66187	0
9	mefenamic acid	formoterol	C0311394	difficulty in walking	13.51428	0
10	cefpodoxime	chlorpheniramine	C0034063	lung edema	13.4577	0
11	9-hydroxyrisperidone	adenosine	C0015967	body temperature increased	13.39606	0
12	dexmedetomidine	perphenazine	C0036572	convulsion	12.31172	0
13	anastrozole	Implanon (Etonogestrel)	C0021311	infection	12.27743	0
14	naratriptan	DCF (docetaxel, cisplatin and 5-fluorouracil)	C0041657	loss of consciousness	11.87367	0
15	cefditoren	loteprednol etabonate	C0027497	nausea	11.83031	0

### 3.2 Baseline characteristics of patients

A total of 268,182 patients who visited Asan Medical Center had the first prescription date for each medication from January 1, 2000, to December 31, 2020, were over 18 years of age, and took cefpodoxime or chlorpheniramine orally were included in the study. After applying the exclusion criteria, the final study cohort consisted of 259,568 patients, including 54,043 patients with a history of taking cefpodoxime, 203,897 with a history of taking chlorpheniramine, and 1,628 with a history of taking both drugs simultaneously (Fig. 2).

To select patients who were taking cefpodoxime and chlorpheniramine concurrently, the duration of medication use was calculated for each patient, and only patients with overlapping durations of cefpodoxime and chlorpheniramine were included in the concurrent medication group. The dosing periods of the cefpodoxime and chlorpheniramine groups are shown in Fig. 3.

Table 2 summarizes the baseline characteristics of the study population. There were no significant differences in sex and the prevalence of cardiovascular disease between the cefpodoxime & chlorpheniramine-treated group and the cefpodoxime-treated group. However, there were differences in age, the prevalence of type 2 diabetes, hypertension, hyperlipidemia, and Pneumonia (p<0.001). And there were no significant differences in the prevalence of hyperlipidemia, and cardiovascular disease between the cefpodoxime & chlorpheniramine-treated group and the chlorpheniramine-treated group. However, there were differences in age, sex, the prevalence of type 2 diabetes, hypertension and Pneumonia (p<0.001).

**Table 2:** Baseline characteristics.

Characteristics	Cefpodoxime & Chlorpheniramine (n = 1,628)	Cefpodoxime & No Chlorpheniramine (n = 54,043)	p value	Cefpodoxime & Chlorpheniramine (n = 1,628)	No Cefpodoxime & Chlorpheniramine (n = 203,897)	p value
Age, mean years (SD)	68.81 (15.29)	63.50 (16.32)	<0.001	68.81 (15.29)	64.17 (16.24)	<0.001
Sex						
Male (%)	839 (51.54)	29308 (54.23)	0.034	839 (51.54)	82026 (40.23)	<0.001
Female (%)	789 (48.46)	24735 (45.77)		789 (48.46)	121871 (59.77)	
Type 2 diabetes (%)	353 (21.68)	7987 (14.78)	<0.001	353 (21.68)	29958 (14.7)	<0.001
Hypertension (%)	620 (38.08)	13979 (25.87)	<0.001	620 (38.08)	58436 (28.7)	<0.001
Hyperlipidemia (%)	198 (12.16)	4284 (7.93)	<0.001	198 (12.16)	20960 (10.3)	0.014
Cardiovascular disease (%)	11 (0.68)	202 (0.4)	0.082	11 (0.68)	1225 (0.6)	0.819
Pneumonia (%)	439 (26.97)	2635 (4.88)	<0.001	439 (26.97)	11378 (5.6)	<0.001

### 3.3 Clinical outcomes

Table 3 provides a summary of the total number of patients, person-years, outcomes, and incidence rate per 100,000 person-years (95% CI) for each of the three groups. In terms of incidence rate per 100,000 person-years, the incidence of lung edema was higher in the cefpodoxime & chlorpheniramine-treated group than in the other groups.

**Table 3:** Incidence rate of lung edema.

	Cefpodoxime & Chlorpheniramine	Cefpodoxime & No Chlorpheniramine	No Cefpodoxime & Chlorpheniramine
Patients	1,628	54,043	203,897
Person-years	17,137	597,351	2,217,300
Outcomes	26	194	1028
Incidence rate per 100,000 PY^1^	151.72	32.48	46.36
95% CI^2^	99.11 - 222.30	28.07 - 37.38	43.57 - 49.29

1 PY: person-years.

2 CI: confidence interval.

### 3.4 Risk of polypharmacy

**Figure 4:**
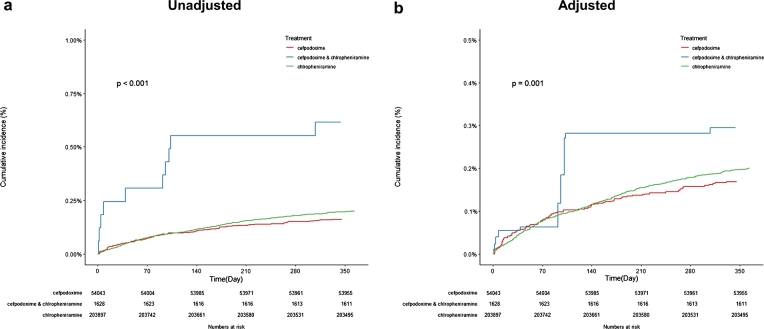
Cumulative incidence of lung edema.

The 1-year cumulative incidence of lung edema in the patient group taking cefpodoxime and chlorpheniramine concurrently was significantly higher than the 1-year cumulative incidence of lung edema in the group taking either medication alone (p=0.001, Fig. 4).

Before adjustment, patients taking cefpodoxime and chlorpheniramine simultaneously had an increased risk of lung edema [hazard ratio (HR) 4.53, 95% CI 3.01-6.82, p<0.001] compared with patients taking cefpodoxime alone. Additionally, patients taking cefpodoxime and chlorpheniramine simultaneously had an increased risk of lung edema (HR 3.23, 95% CI 2.19-4.76, p<0.001) compared to patients taking chlorpheniramine alone.

After adjustment, patients taking cefpodoxime and chlorpheniramine simultaneously had around two times the risk of lung edema compared to patients taking cefpodoxime alone [hazard ratio (HR) 2.10, 95% CI 1.26-3.52, p<0.005]. Patients taking cefpodoxime and chlorpheniramine simultaneously experienced a 64% increased risk of lung edema (HR 1.64, 95% CI 0.99-2.69, p=0.05) compared to patients taking chlorpheniramine alone. These cox-proportional hazard ratios are shown in Table 4.

**Table 4:** Hazard ratio.

		HR^1^	95% CI^2^	p-value
cefpodoxime & chlorpheniramine vs. cefpodoxime - lung edema	Unadjusted	4.53	3.01-6.82	<0.001
	Adjusted	2.10	1.26-3.52	<0.005

cefpodoxime & chlorpheniramine vs. chlorpheniramine - lung edema	Unadjusted	3.23	2.19-4.76	<0.001
	Adjusted	1.64	0.99-2.69	0.05

1 HR: hazard ratio.

2 CI: confidence interval.

The results showed that taking cefpodoxime and chlorpheniramine simultaneously increased the hazard ratio of lung edema by 2.1 times compared to cefpodoxime alone (p<0.005) and by 1.64 times compared to chlorpheniramine alone (p=0.05).

## 4 Discussion

To our knowledge, this is a retrospective cohort study that successfully validated a specific drug-drug side effect predicted by deep learning using RWD. This study was conducted to validate the potential polypharmacy side effects of DDIs through a deep learning-based prediction utilizing public data. There are many methods for predicting side effects of DDIs based on deep learning, but there are few verified studies as RWD, so it is difficult to use them in the medical field. To apply a polypharmacy side effects prediction system using deep learning in the medical field, it is necessary to confirm these predictions with RWD. We chose the DeSIDE-DDI method for validation, which has been shown to have better performance for prediction than previous methods. We had data from a large tertiary general hospital called Asan Medical Center, which is an acceptable cohort for this kind of population-based research. Accordingly, we conducted an analysis by checking the list of unknown drug-drug-side effect combinations predicted through a deep learning-based polypharmacy side effects prediction model and comparing it with cases of simultaneous use of the two drugs found through actual patient data. The results showed that patients who had taken cefpodoxime and chlorpheniramine simultaneously had a higher risk of developing lung edema than patients who had taken cefpodoxime or chlorpheniramine alone.

Cefpodoxime is an antibiotic used as a treatment for various bacterial infections and is also known to be used in cases of lung edema caused by bacteria [21]. Chlorpheniramine is an antihistamine used to treat allergic reactions [22], with a known case report of pulmonary edema after taking chlorpheniramine [23]. Recently, a study showed that cefpodoxime proxetil could attenuate Ca^2+^ influx in SH-SY5Y cells [24]. Calcium channel blockers have been known to cause edema [25], and since both drugs are associated with the regulation of intercellular calcium levels, there may be some unknown relationships between calcium channel blockers and the two drugs.

In previous studies, the difference between the predicted drug-drug-side effect relationship and the prevalence of side effects occurring in actual patients' clinical data was analyzed through prospective studies [26], [27]. While some researchers have performed retrospective cohort studies on potential drug-drug interactions, most of these studies have only confirmed the prevalence of side effects occurring in cohorts of interest [28], [29], [30]. In this study, we conducted an exploratory retrospective investigation without specifying a particular cohort to evaluate the potential of real-world patient data in identifying adverse events for unknown drug-drug interactions. Despite the artificial intelligence predicting an absence of side effects when two drugs were co-administered, we verified instances where side effects were observed. This highlights the critical role of real patient data in validating the adverse event prediction algorithm for polypharmacy using artificial intelligence.

*Limitations*

This study has several limitations. First, the analysis was conducted using patient data from a single country and institution. Since the RWD of a single country and a single institution was analyzed, it is unknown whether similar results can be obtained if the RWD of multiple nations and institutions are analyzed. Second, potential unrecognized covariates may exist. Although we selected patients to be representative of general demographic characteristics, there may be unrecognized covariates, such as other lung edema-related diseases or medicines. Third, the exact mechanism of lung edema caused by the co-administration of cefpodoxime and chlorpheniramine remains unknown. This issue requires further investigation and resolution.

Despite these limitations, it is beneficial to validate these polypharmacy side effect predictions using RWD because it can be a new way of conducting trials without harm. Also, demands a platform capable of statistical analysis that deep learning can verify unknown drug-side effects using patient data have increased [31], [32], [33]. Therefore, it is important to find and confirm drug combinations that are likely to occur in the real world. In this study, there were many cases where the drug combinations predicted by deep learning were not used. To complement these predictions of polypharmacy side effects, new datasets of frequently used drugs and relatively serious diseases will be needed.

## 5 Conclusion

We validated the polypharmacy side effect prediction by using RWD. In patients who had taken cefpodoxime and chlorpheniramine simultaneously, the long-term risk of lung edema was higher than that of the patients who had taken only cefpodoxime or chlorpheniramine.

## Ethics declarations

This study was reviewed and approved by the Institutional Review Board of Asan Medical Center, conducted in accordance with the 2008 Declaration of Helsinki, with the approval number: 2021-0303.

## CRediT authorship contribution statement

**Gaeun Kee:** Writing – review & editing, Writing – original draft, Formal analysis, Conceptualization. **Hee Jun Kang:** Formal analysis. **Imjin Ahn:** Formal analysis. **Hansle Gwon:** Formal analysis. **Yunha Kim:** Formal analysis. **Hyeram Seo:** Formal analysis. **Heejung Choi:** Formal analysis. **Ha Na Cho:** Formal analysis. **Minkyoung Kim:** Formal analysis. **JiYe Han:** Formal analysis. **Seohyun Park:** Formal analysis. **Kyuwoong Kim:** Writing – review & editing. **Tae Joon Jun:** Writing – review & editing, Supervision, Conceptualization. **Young-Hak Kim:** Writing – review & editing, Supervision, Conceptualization.

## Declaration of Competing Interest

The authors declare that they have no known competing financial interests or personal relationships that could have appeared to influence the work reported in this paper.

## Data Availability

Data will be made available on request. Data are available from Asan Medical Center and are available upon reasonable request due to ethical concerns and confidentiality agreements.
